# TB preventive therapy preferences among children and adolescents

**DOI:** 10.5588/ijtld.22.0645

**Published:** 2023-07-01

**Authors:** M. Strauss, D. T. Wademan, A. Mcinziba, G. Hoddinott, M. Rafique, L. N. Jola, C. Streicher, K. du Preez, M. Osman, J. Boffa, H. Hausler, A. C. Hesseling, Y. Hirsch-Moverman

**Affiliations:** 1 Health Economics and HIV and AIDS Research Division (HEARD), University of KwaZulu-Natal, Durban, South Africa; 2 Desmond Tutu TB Centre, Department of Paediatrics and Child Health, Faculty of Medicine and Health Sciences, Stellenbosch University, Cape Town, South Africa; 3 School of Human Sciences, Faculty of Education, Health and Human Sciences, University of Greenwich, London, UK; 4 TB Think Tank, The Aurum Institute, Johannesburg, South Africa; 5 Centre for Rural Health, University of KwaZulu-Natal, Durban, South Africa; 6 TB HIV Care, Cape Town, South Africa; 7 Department of Family Medicine, Faculty of Health Sciences, University of Pretoria, Pretoria, South Africa; 8 ICAP at Columbia University, New York, NY, USA

**Keywords:** tuberculosis preventive therapy, discrete choice experiment, patient-centred care, children, preferences

## Abstract

**BACKGROUND:**

TB preventive therapy (TPT) is critical for ending TB, yet implementation remains poor. With new global guidelines expanding TPT eligibility and regimens, we aimed to understand TPT preferences among children, adolescents and caregivers.

**METHODS:**

We undertook a discrete choice experiment among 131 children, 170 adolescents and 173 caregivers, and conducted 17 in-depth interviews in 25 clinics in Cape Town, South Africa. The design included attributes for location, waiting time, treatment duration, dosing frequency, formulation/size, side effects, packaging and taste. Mixed-effects logistic regression models were used for analysis.

**RESULTS:**

Among children and caregivers, the number and size of pills, taste and side effects were important drivers of preferences. Among adolescents and caregivers, clinic waiting times and side effects were significant drivers of preferences. Adolescents expressed concerns about being stigmatised, and preferred services from local clinics to services delivered in the community. Dosing frequency and treatment duration were only significant drivers of choice among adolescents, and only if linked to fewer clinic visits.

**CONCLUSIONS:**

Introducing shorter TPT regimens in isolation without consideration of preferences and health services may not have the desired effect on uptake and completion. Developing TPT delivery models and formulations that align with preferences must be prioritised.

## Abstract (fr)

**CONTEXTE:**

Le traitement préventif contre la TB (TPT) est essentiel pour mettre fin à la TB, mais sa mise en œuvre laisse à désirer. Avec les nouvelles directives mondiales élargissant l’éligibilité au TPT et les schémas thérapeutiques, nous avons cherché à comprendre les préférences en matière de TPT chez les enfants, adolescents et aidants.

**MÉTHODES:**

Nous avons eu recours à la méthode des choix discrets auprès de 131 enfants, 170 adolescents et 173 aidants, et mené 17 entretiens approfondis dans 25 cliniques du Cap, Afrique du Sud. Le modèle comprenait des attributs pour le lieu, le temps d’attente, la durée du traitement, la fréquence d’administration, la formulation/taille, les effets secondaires, l’emballage et le goût. Des modèles de régression logistique à effets mixtes ont été utilisés pour l’analyse.

**RÉSULTATS:**

Chez les enfants et les aidants, le nombre et la taille des comprimés, le goût et les effets secondaires étaient des facteurs de préférence importants. Chez les adolescents et les aidants, les temps d’attente dans les cliniques et les effets secondaires étaient des facteurs de préférence significatifs. Les adolescents ont exprimé leur crainte d’être stigmatisés et préféraient les services des cliniques locales aux services fournis dans la communauté. La fréquence d’administration et la durée du traitement n’étaient des facteurs de choix significatifs que chez les adolescents, et seulement s’ils étaient liés à une réduction du nombre de consultations à la clinique.

**CONCLUSIONS:**

L’introduction de schémas thérapeutiques plus courts pour le TPT sans tenir compte des préférences et des services de santé pourrait ne pas avoir l’effet escompté sur l’adoption et l’achèvement du traitement. La priorité doit être donnée à la mise au point de modèles d’administration et de formulations de TPT qui tiennent compte des préférences.

TB is one of the leading causes of death globally; and after HIV it is the second highest contributor to disease burden among children aged 5–14 years in South Africa.^1^,^2^ TB preventive therapy (TPT) for the prevention of drug-susceptible TB is highly effective and if implemented effectively, can prevent TB disease and death.^3^

The WHO reported that only 57% of TB-exposed South African children aged <5 years initiated TPT in 2021.^2^ ‘Integrated, patient-centred TB care and prevention’ is the first of three pillars of the End TB strategy and considered essential for the attainment of End TB targets and TB-related Sustainable Development Goals (SDGs).^4^,^5^ Recent interest in providing patient-centred care has sparked an increased emphasis on eliciting patient participation in shared decision-making, particularly when multiple treatment options are available and a clearly superior one is not evident.^6^–^8^ Understanding user preferences regarding TPT regimens and service delivery models is critical to reducing negative impacts on quality of life and improving clinical outcomes and patient experiences.

The WHO recently endorsed the use of shorter TPT regimens for children and adolescents, including a 3-month daily rifampicin and isoniazid (3HR) regimen and a 3-month, once-weekly rifapentine and isoniazid (3HP) regimen.^3^ These regimens greatly reduce the length of treatment compared to the current standard-of-care regimen, the 6-month, daily isoniazid (6H) regimen. We aimed to understand the relative importance of eight attributes, including treatment duration, of TPT service delivery models and drug regimen characteristics among South African children, adolescents and their caregivers.

## METHODS

### Study setting

The study was conducted in 25 primary health care (PHC) facilities across three of the eight sub-districts in the Cape Metropolitan District, Western Cape Province. The Western Cape Province has a particularly high burden of paediatric TB, with children aged <15 years constituting 13% of the total notified TB cases.^9^ In 2021, nearly 25,000 notified TB cases and over 2,000 TB deaths were recorded in the district.^10^ Most TB-affected households are in high-density, low-income, peri-urban townships, informal housing areas and low-cost housing developments. These areas have limited access to municipal services, high rates of crime, and residence is often fluid as people transition in and out pursuing limited opportunities. The TB programme in the districts is nurse-driven - providing TB testing, ongoing treatment of both drug-susceptible and drug-resistant TB, and TPT services at the PHC facility level. The TB programme is supported by community-based health workers (CHWs).

### Study design

We used a discrete choice experiment (DCE) to understand preferences relating to TPT service delivery models and drug regimens. A DCE is a quantitative behavioural economics technique embedded in well-established economic theory, used to elicit information about preferences and key drivers of choice by offering participants hypothetical scenarios (“choice sets”) that force trade-offs between the key characteristics of goods or service.^11^ This methodology has been used to inform health policy in low-income settings, and enables the relative valuation of individual characteristics of health service packages (e.g., location or duration of treatment); it can thus be used to determine the specific characteristics of services of the highest utility to the target population. We supplemented the DCE with qualitative data to contextualise interpretations.

### DCE design and analysis

We followed the general design method for conducting DCEs,^12^ accounting for the choice context and healthcare processes, to select relevant attributes and levels.^13^ In this design, we drew from a previous study^14^ to develop an initial set of attributes and levels which we refined through engagement with key stakeholders (Table 1). The attributes and their levels were each deemed reasonable fits with how TB services and regimens may be implemented in the local setting (Table 1).

**Table 1:** Attributes and levels included in the discrete choice experiment design

Attribute	Level 1	Level 2	Level 3	Level 4
Health system attributes				
Location	Local clinic^*^	CHW home visit	Community centre	Mobile clinic
Wait time in the clinic	15 min	45 min^*^	1 h 30 min	3 h
Drug regimen attributes				
Duration of treatment and visit frequency	6 monthly visits for 6 months^*^	6 months, 1 visit	3 monthly visits for 3 months	3 months, 1 visit
Dosing frequency	Once a day^*^	Once every 2 days	Once a week	Once ever
Formulation and size of pills	2 small pills^*^	6 small pills	2 medium pills	Dissolvable
Side effects	No side effects^*^	Side effects that only you will notice	Side effects that will be obvious to other people who have had TB treatment	Side effects that will be obvious to anyone
Packaging	Non-discreet/noisy packaging^*^	Discreet/quiet packaging	—	—
Taste	Not bitter^*^	Bitter	—	—

^*^ Baseline levels used for analysis.

CHW = community healthcare worker.

A fractional factorial, main effects design was used, and choice sets were designed to ensure zero overlap of attributes in each choice set and level balance.^15^ Thirty-two binary choice sets were generated and blocked into four versions such that each participant was presented with eight choice sets from the full design. Fieldworkers presented illustrated choice sets using booklets, with one choice set per page. A binary, unlabelled approach was used, so that in each choice set, participants were asked to choose between two alternatives (“Option A” and “Option B”) – a design to reduce cognitive burden especially important for child and adolescent participants. Stata v15 (Stata, College Station, TX, USA) was used to generate a statistically optimal design,^16^ using the D-efficiency criterion.^15^,^17^,^18^

The study tool was piloted with fieldworkers and study staff to ensure that the task was easily understandable, and that the level of complexity of the questionnaire was not too cognitively burdensome. Some of the instructions, definitions of the attributes and levels were altered to improve clarity of understanding. Once the tools had been finalised, they were piloted in the field among a range of caregivers, adolescents and children, both to test that the design of the DCE instruments was well aligned to the local context and study settings, as well as the fieldwork processes and procedures. This piloting exercise also provided fieldworkers an opportunity to practice delivering the instructions and definitions before beginning data collection with the study respondents.

Three groups of participants were recruited for the DCE: children (aged 8–14 years); adolescents (aged 15–19 years); and caregivers aged ≥18 years providing care to a child aged <15 years. Xhosa, Afrikaans or English-speaking patients receiving health services at a study site, or their caregivers who were not currently on treatment were eligible for inclusion in the study. Caregivers and children were actively recruited with the assistance of facility staff who referred them to the study during routine visits. Using an established general principle in the DCE literature,^19^ we recruited a minimum sample size of 125 respondents per stratification.

Analyses were conducted in STATA using fixed-effects logit models (not presented here), followed by mixed-effects logit models as the primary tools for estimation of parameters. Results were presented as attribute-level specific coefficients in comparison to a baseline scenario – most closely aligned with standard of care in the Western Cape (Table 1). Stratified mixed-effects logit models using Halton draws with 1,000 replications were used to estimate both a mean effect and standard deviation (SD) for each of the effects across each group (children, adolescents and caregivers). The larger the SD estimate, the greater the variability in preferences across respondents. The significance of heterogeneity estimates is shown by the significance of the 95% confidence intervals (CIs).

### Qualitative design and analysis

Two types of qualitative data were included to improve the explanatory power of the DCE – qualitative field notes and in-depth interviews. Graduate socio-behavioural scientists completed daily semi-structured field note forms (*n* = 180 forms from ~12 weeks of DCE administration by four fieldworkers to 464 participants) to reflect on their interactions with participants.

For the in-depth interviews, 5 health workers experienced in TPT provision, 2 children and 5 adolescents with TPT experience, and 5 caregivers with experience administering TPT to their children (17 participants in total) were sampled purposively for diversity in sex and age. Interviews lasted 50–75 min and topics covered included getting to know the participant, TPT experiences and TPT preferences. Interviews were audio-recorded, and fieldworkers took unstructured hand-written field notes. Detailed case descriptions were written by fieldworkers within 48 h of the interview, while listening to the audio recording and reviewing field notes, and discussed with the study Principal Investigator. The study team compared case descriptions, highlighting common and divergent themes.

### Ethics

The protocol was approved by Stellenbosch University’s Health Research Ethics Committee (N20/10/110), Tygerberg, and the City of Cape Town Health Directorate, Cape Town, South Africa. All participants aged 8–14 years provided written assent with their caregiver providing written informed consent. All participants aged ≥15 years provided written informed consent.

## RESULTS

### DCE participant profile

Most DCE participants were female, HIV-negative and had no prior TB treatment experience. The mean age of children, adolescents and caregivers was respectively 10, 17 and 32 years (range 18–64 years; Table 2).

**Table 2:** DCE participant profile

		Children (8–14 years) (*n* = 131) *n* (%)	Adolescents (15–19 years) (*n* = 170) *n* (%)	Caregivers (*n* = 173) *n* (%)
Sex	Female	78 (60)	150 (88)	151 (87)
	Male	52 (40)	16 (9)	22 (13)
	Other	1 (1)	4 (2)	0 (0)
HIV status	HIV-positive	5 (4)	12 (7)	10 (6)
	HIV-negative	126 (96)	158 (93)	163 (94)
Previous TB treatment	Ever	3 (2)	5 (3)	19 (11)
	Never	124 (95)	165 (97)	151 (87)
Age, years, mean 6 SD		10 6 2	17 6 1	32 6 10

DCE = discrete choice experiment; SD = standard deviation.

### Preferences in health system attributes

**Figure 1:**
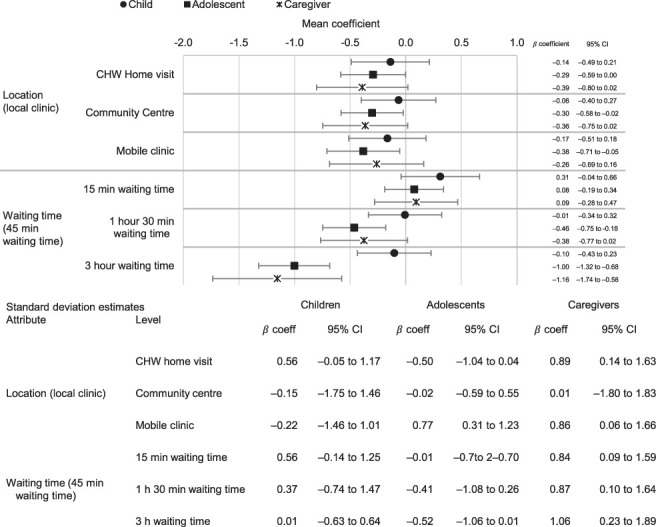
Stratified mixed effects logit mean coefficient and standard deviation estimates: health system attributes. CI = confidence interval; CHW = community healthcare worker.

Figure 1 shows the results of the stratified mixed-effects model for location of services and waiting time. Overall, preference structures show a similar pattern regarding location of services, with a preference for services delivered at a local clinic. These results are only statistically significant for adolescents; in the qualitative data (Table 3), children and caregivers explained that location was not especially important to their choices. Generally, adolescents stated a preference for receiving TPT at their local clinic because people attend clinics for a variety of reasons, and they would not be identified as needing a TB service. Where possible, adolescents would want to avoid TB-associated stigma and not receive home visits. Furthermore, taking TPT in the local clinic facilitated their access to services such as diagnostic testing that are important for identifying other health-related issues (Figure 1).

**Table 3:** Children, adolescents, caregivers, and health workers preferences of health system and drug regimen attributes from qualitative interviews

Domain	Attribute	Participant	Illustrative quote
Health system attributes	Location	Children	“I’d choose to go to the clinic because at the clinic they won’t make a mistake of giving me wrong medication [...] no one else will know why I’m there. People go to clinic for different things” (age 10, female)
		Adolescents	“Some people at home don’t know I’m taking pills. So, for CHWs to deliver it home will lead to unintentional disclosure. For example, I was advised not to disclose to my grandmother because we could talk about this at home and she goes around telling my aunties and stuff” (age 15, female)
		Caregivers	“For now, I see nothing wrong for fetching treatment from the local clinic because they do other things as well [...] checking and running other tests” (age 47, female)
		Health workers	“To make things easier, the CHWs should make home visits because the patients don’t want to [... come] to the clinic” (age 46, female).
	Waiting time	Children	“I want 15 min because people don’t take pleasure to sit at the clinic longer because even the chairs are cold” (age 13, male)
		Adolescents	“15 min would be better [...] staff members are slow, and they have a potential to work faster but [...] I could wait for more than 15 min [...] it is my health, my life, so I’d still wait” (age 18, female)
		Caregivers	“15 min would be nice but if the situation forces me, I can even wait 3 h as long as the health workers inform us of what’s happening because some people are impatient, they end up going back home when they feel neglected” (age 47, female)
		Health workers	“In a perfect world 15 min would be great, but the way clinics are so packed, there is no way you can see someone and give services to each for 15 min, at least 1 h and 30 min to 3 h maximum is fine” (age 36, female)
Drug regimen attributes	Formulation of medication and size of pills	Children	“I don’t like swallowing lots of pills. Even when I have fever, my mom would give me her headache pills, but I dislike them” (age 9, male)
		Adolescents	“I prefer dissolvable pills because you see, I [...] take one big pill (ARVs) every morning so it’s going to be hard for me to swallow other pills on top of that” (age 16, male)
		Caregivers	“I am cool with any size or number of pills but for my daughter I’d prefer the two small ones [...] she is still young you know, but I think the bigger ones could be more effective compared to the small ones” (age 33, female)
		Health workers	“I would prefer 2 small pills because they would be easier to swallow especially for children or the dissolvable ones for as long as they don’t taste bitter” (age 35, female)
	Side effects	Children	“What’s the point to take medication that will make me feel sick or drowsy? Pills with side effects seen by people will make ‘township journalists’ [gossipers] think I have HIV” (age 13, female)
		Adolescents	“There rather be no side effects, if I experience side effects maybe I feel dizzy it will affect the days when I have to go to school [...] If I were to write a test or exam and I feel dizzy they’ll send me home and I could fail” (age 16, male)
		Caregivers	“I would prefer no side effects [but] no treatment is without side effects and therefore side effects only I would notice could be better because it’s also an indication that the medication is working [effective]” (age 43, female)
		Health workers	“No, no side effects or side effects only a patient would notice [because] side effects obvious to other people would be associated with stigma which is the main barrier to adherence” (age 37, female)
	Taste of medication	Children	“I want very very small pills or dissolvable ones that will taste sweet like those yellow painkillers so when I drink them, they taste like juice” (age 8–14 years, no sex given)
		Adolescents	“I am used to medicine [ARVs]. Personally, I am someone who strongly believes in traditional medicine and if it’s not bitter then that’s horrible for me. Have you tasted a plain yogurt? It’s terrible” (age 17, female)
		Caregivers	“I prefer the bitter pills because I have a belief that once something tastes bitter, it’s efficient compared to something that doesn’t have a taste” (age 43, female)
		Health workers	“I prefer non-bitter tasting pills that would rather taste like a syrup, especially children, they dislike bitter pills which makes it hard for them to ‘enjoy’ taking tablets” (age 36, female)
	Duration of treatment and visit frequency	Children	“It’s better to do one clinic visit and take treatment for 3 months because I don’t want to miss school days because I have to come here [clinic] more often” (age 14, male)
		Adolescents	“I choose 1 clinic visit for 3 months treatment because a monthly visit will annoy me [...] And 1 visit for 6 months treatment? Oh, no that whole package would be chaotic and I might find some of those pills laying around at home, because it would be a lot” (age 17, male)
		Caregivers	“Coming every now and then to the clinic is right, perhaps there is something the doctor might detect or see from you that you wouldn’t notice” (age 33, female)
		Health workers	“It’s fewer [pills] and less duration for me. Some people are working and won’t have time to come to the clinic every month, they would need to miss work but get in trouble with their bosses” (age 37, female)
	Dosing frequency	Children	“I prefer to have them [pills] every day [...] if you skip other days or doses they might not work right [effectively] but I know my friends would choose to eat them once ever because they don’t like pills” (age 10, female)
		Adolescents	“If the pill could be dissolvable, I’d prefer to take it daily because how could it work effectively if I take it once ever ... besides there’s a reason why the nurses advise that we should take it every day” (age 17, female)
		Caregivers	“If you skip a day or days, by the time you drink the pill again for example, the last one you ate is no longer active in your body” (age 43, female)
		Health workers	“If the treatment was sufficient, I’d take the option whereby the patient takes the medication once ever [...] we are not there yet as a country because I’d take ‘once a week’ [because] it would be better for the patient to manage” (age 44, female)
	Packaging	Children	“I don’t want noisy [packaging] because it could cause [anxiety] to people who have stress here in the house” (age 10, female)
		Adolescents	“I choose discreet packaging for me [...] sometimes when I leave home to the clinic, I tell people at home that I’m going to the supermarket. Now everyone will notice that I’m coming from the clinic because I’m carrying this huge noisy container” (age 18, female)
		Caregivers	“You see the noisy packaging will lure ‘*tsotsis*’ [gangsters] because people know these tablets and gangsters use them for smoking” (age 43, female)
		Health workers	“Patients are currently complaining about ARVs that they are making noises and some patients even take from my tissue and wrap the pills inside to contain the noise. So discreet package is the right one” (age 35, female)

CHW = community health worker; ARV = antiretroviral.

Participants preferred shorter waiting times; however, these findings were only significant for adolescents and caregivers, and particularly for very long waiting times (3 hours). In qualitative interviews, most participants reported that they preferred waiting times to be 15 min, but this would be unlikely in overcrowded PHC facilities. Therefore, participants were mostly willing to accept longer waiting times and rather prioritise other attributes. Generally, adolescents who reported not wanting to wait long in facilities indicated that they did not want to be seen by friends or family who might ask them why they were there (Table 3).

### Preferences in drug regimen attributes

**Figure 2:**
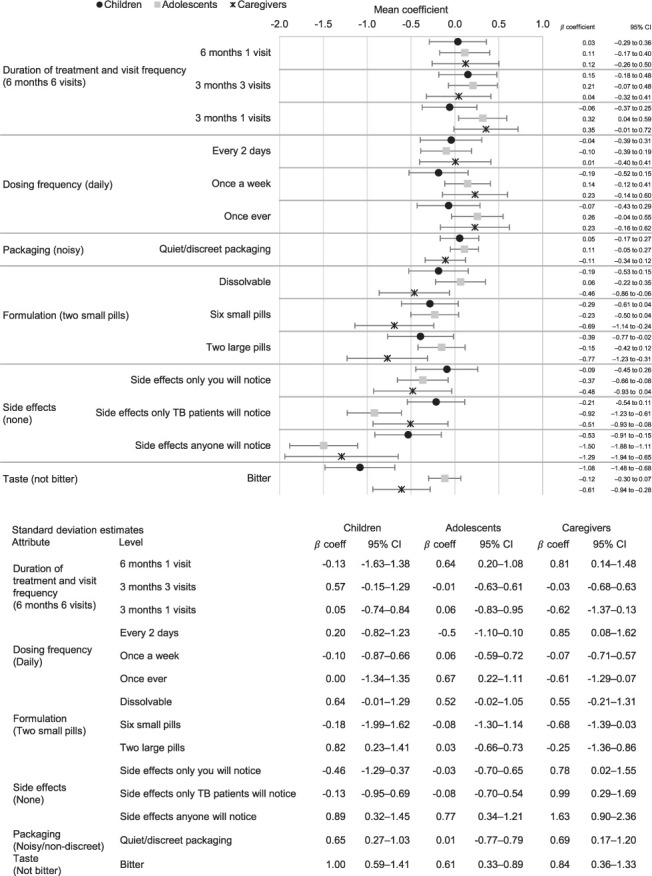
Stratified mixed effects logit mean and standard deviation estimates: drug regimen attributes. CI = confidence interval; CHW = community healthcare worker.

Figure 2 shows the results of the stratified mixed-effects model for duration of treatment and visit frequency, dosing frequency, packaging, medication formulation, side effects and medication taste. These attributes were found to have the most significant effect on preferences across the three groups. Adolescents were indifferent about pill size and formulation, while children were significantly less likely to choose alternatives with two large pills compared to alternatives with two small pills. Caregivers mostly preferred formulations with two small pills, being less likely to choose alternatives with six small pills, two large pills or dissolvable formulations (all highly significant). Qualitative findings suggested that children and adolescents who reported preferring to take either two small pills or dissolvable pills were concerned that two large pills or six small pills might get stuck in their throat. Those who were also on antiretroviral therapy said that additional pills would be a burden. Caregivers and health workers preferred two small pills or dissolvable pills because they are easier to administer but some reported a perception that fewer or smaller pills may be less effective at preventing TB (Figure 2).

Taste had a strong and highly significant effect on preferences for children and caregivers, who preferred formulations that were not bitter, while adolescents had no clear preference regarding taste. In interviews, adolescents, caregivers and health workers noted an association between medication, including traditional medicine, tasting bitter and its perceived effectiveness. In contrast, children strongly preferred sweet tasting pills, which they said would make it much easier to adhere and cause far less tension with their caregivers or health workers who are supporting their adherence.

Side effects had a significant effect on preferences across all groups, but particularly for adolescents and caregivers. Qualitative findings suggest that participants expected that all medicines could have side effects. Thus, although they would prefer fewer and less severe side effects, they accepted some side effects. Despite acknowledging the significance of avoiding TB-related stigma, many participants did not perceive the side effects of TPT as distinctive enough to reveal their TB status to others. Nevertheless, some adolescents emphasised that any noticeable side effects would undermine their adherence to TPT, as they would be less likely to take it consistently to avoid drawing attention to their TB status.

There was an overall pattern showing a possible preference for shorter treatment duration and fewer clinic visits, but this was only significant among adolescents who preferred 3 months of treatment with just one clinic visit compared to 6 months of treatment with monthly clinic visits. In interviews, adolescents and children explained that fewer clinic visits and shorter duration were preferable because going to clinic sometimes clashed with school days. However, caregivers and health workers said monthly clinic visits were useful for adherence support, additional care and diagnostic tests, if needed.

Dosing frequency was not found to be a significant driver of choice across any groups in this study in relation to the other attributes included in this design. In interviews, participants indicated a concern that not taking a pill every day may cause them to forget to take their pills. They also reported concerns that pills taken less frequently may also be less effective. Health workers suggested that shorter duration of regimen is more important than reducing dosing frequency. Overall, most participants were in favour of less frequent dosing, but not if it came at the cost of other attributes.

Packaging was not a significant driver of choice for any of the groups. However, heterogeneity in this attribute suggests packaging may be an important consideration for some caregivers and children. In qualitative interviews, children and adolescents reported that noisy packaging presents a challenge to privacy, and that having discreet packaging could allow them to take their medication anywhere and at any time without anyone noticing. They reported that this could strengthen their uptake of TPT medication in future.

### Heterogeneity

Children’s preference structures were largely homogeneous across attributes and levels, apart from preferences for large pills compared to small pills, side effects that anyone could notice, packaging and taste. For adolescents, there was a high degree of heterogeneity around taste, the number of clinic visits, location, dosing frequency and the importance of having medications with side effects that are not noticeable to others. Caregivers’ trade-offs were more complex than children’s and adolescents’, and we found more heterogeneity, particularly regarding packaging, location and side effects. The sample size within groups in this study did not allow for further investigation of preference heterogeneity but is an important question for future study.

## DISCUSSION

We found that children prefer TPT regimens that have smaller pills, with no side effects and which are not bitter. Adolescents prefer TPT regimens that do not require community-based care, short waiting times, shorter regimens with fewer clinic visits and no side effects. Caregivers prefer TPT regimens that are easy to prepare and administer, shorter waiting times and with no side effects. We found a general pattern of a preference for fewer and smaller pills, and none of the groups considered dissolvable formulations to be significantly preferable to formulations with two small pills.

Overall, children’s preference structures were relatively simple, with only a few attributes showing significant results, including formulation, side effects and taste. Adolescents had more complex trade-off structures than younger children and were more concerned about health system attributes, being noticed taking treatment and experiencing stigma over drug regimen attributes such as formulations, packaging and taste. Caregivers had the most complicated trade-off strategies, as well as a higher degree of heterogeneity among more of the attributes and levels than adolescents or children. Caregivers appeared to accept existing health systems attributes but emphasised a need for improved drug regimen attributes, preferring fewer, smaller pills, with no side effects.

Taste was important to children and caregivers, but not significantly so for adolescents, although there was a high degree of heterogeneity regarding taste, particularly among caregivers and adolescents. This is likely to be an indication that for younger children, younger adolescents and caregivers of younger children, taste has a large effect on preferences, which will in turn impact uptake and adherence. However, older adolescents are likely to be more willing to trade off medication taste with other attributes of drug regimens and service delivery models. This was similarly the case regarding pill size and formulation, and the heterogeneity here suggests that some of the older children in the sample were not as concerned about the size of the pills. Our findings are similar to those found in a previous DCE among children, their caregivers and healthcare providers carried out in Eswatini – taste had the greatest effect on preferences; pill count and pill size was an important driver of preferences; shorter waiting times were significantly preferred to longer waiting times; and treatment duration only had a significant effect when this was combined with fewer clinic visits.^14^ While dosing frequency was not identified as an important driver of preferences among children in either of the studies, caregivers and healthcare providers in Eswatini had a significant preference for less frequent dosing schedules.^14^

Our study combined qualitative and quantitative methods, including perspectives of targeted TPT users (children and adolescents), caregivers and health workers, strengthening the study findings. This is the first study of its kind in South Africa. Although we used a DCE design with no opt-out option, which limits inferences to actual demand for services, we believe this design choice is appropriate as it maximises the amount of trade-off information. This study was limited to the Western Cape, South Africa. The Western Cape’s health system is not representative of other settings, including other settings in South Africa, limiting the generalisability of our findings and our ability to directly compare with findings from other settings. However, we purposively selected the Western Cape as one of the provinces with a high burden of TB and strong programmes that could support TPT delivery for children and adolescents. In addition, we did not have a random sample as it is difficult to get a true random sample in this context in terms of the size and spread of the population from which our participants were drawn. We therefore sent fieldworkers out on different days and at different times to mitigate the non-randomness of the sample. We note that it is useful to compare the preference structures of targeted users in our study with studies from elsewhere in the world (and specifically in the southern African region). These comparisons must be made with the understanding that the South African context is marked by the legacy of the Apartheid government characterised by a fragmented and inequitable health system, which has resulted in varying types and forms of generational trauma and varying levels of mistrust of the government-run health system. Most adolescents and caregivers in our sample were female, which may limit the generalisability of our findings; however, this sample bias may also be a relatively accurate reflection of the population accessing healthcare services at community clinics.

Our findings are similar to those reported among caregivers of young children in Lesotho, who reported wanting a shorter treatment duration with fewer pills per dose – although some caregivers reported concerns about their ability to remember once-weekly dosing.^20^ Although shorter TPT regimens – isoniazid and rifampicin once daily for 3 months (3HR) – have been found efficacious with reportedly higher adherence rates compared to 6 months of daily isoniazid,^21^,^22^ our findings suggest that the effect of shorter regimens on preferences is only likely to be significant if combined with fewer clinic visits. A small exploratory Peruvian study found that among caregivers of children exposed to TB in the household, having a formulation that was considered “child-friendly” was more important than regimen duration.^23^ This is consistent with the findings in our study, which suggests that fewer, smaller pills and non-bitter formulations have a greater effect on preferences than shorter regimens.

Unlike our findings, a multi-national study revealed treatment side effects only marginally impacted caregivers’ willingness to initiate a newly developed TPT for multidrug-resistant TB (MDR-TB)^24^ – our findings, however, may not be directly comparable as the focus was on drug-susceptible TB, and MDR-TB patients may have different preference structures. A previous study of potential barriers to TPT implementation among South African caregivers reported a similar finding to our analysis of adolescents – that fear of stigmatisation associated with having TB was a greater hindrance to TPT adherence than the treatment formulation, daily administration or treatment side effects.^25^ In a Rwandan-based study reporting high levels of TPT adherence (88%), caregivers noted socio-economic, structural and health systems barriers as the greatest impediment to TPT uptake and adherence.^26^ Studies of TPT uptake among children and adolescents also reported socio-economic circumstances, lack of knowledge and limited access to health systems as barriers to adherence in Brazil and Indonesia.^27^,^28^

Caregivers of children aged <5 years exposed to MDR-TB in Cape Town reported high levels of acceptability of a taste-masked, dispersible formulation of levofloxacin.^29^ A subsequent qualitative study of this formulation in Cape Town reported high levels of caregiver acceptability due to ease of preparation, administration and suitable palatability, with less common situational factors resulting in impediments to management for some caregivers.^30^ In contrast to caregiver and adolescents’ preferences for clinic-based care in our study, several studies report higher adherence rates when TPT is delivered at the community- or household-level.^31^,^32^ Although shorter preventive therapy, including 3HP, 3HR and 1HP, have been proven efficacious in children, attention to the development of formulations that align with children’s preferences – including taste-masking, and reducing the number and size of pills (and dispersible formulations for very young children) for these regimens – is still lagging,^22^,^33^–^35^ as are the implications on effectiveness if doses are missed or shared among household members.

## CONCLUSIONS

To enhance patient-centred healthcare delivery, it is crucial to make TPT (user-friendly and comprehensively explained. This will not only facilitate uptake and adherence but also foster trust among end-users towards new treatment options. This must include clear communication regarding proven effectiveness, regimen options and potential side effects. Research on the implications of missing or sharing doses of shorter regimens in adolescents and children is also a priority. More palatable formulations for children and caregivers of young children remains a priority and requires clearer guidance on how to use food or flavour agents to taste mask until such formulations are developed.

The diversity in preferences among children, adolescents and caregivers suggests that there will be no one-size-fits-all preferred regimen and means that switching to shorter regimens or requiring fewer clinic visits is, in isolation, unlikely to have a significant effect on TPT uptake or adherence. This is important for understanding the likely changes in demand for TPT when new regimens are rolled out. Rather, choices between available TPT regimens should be offered so the person initiating TPT (who will often share a household with a TB patient) can make tailored choices. Where tailored options such as home-based or alternative collection points are not offered, a fast-lane for adolescent collection could be considered. At the same time, participants valued the opportunity to interact with health workers and receive adherence support and related care. Further developing family-centred, integrated approaches to identification and screening, TPT initiation and adherence support between community-based and facility-based health workers is important to capitalise on the opportunities of newer regimens.
